# Metformin Ameliorates Diabetic Cardiomyopathy by Activating the PK2/PKR Pathway

**DOI:** 10.3389/fphys.2020.00425

**Published:** 2020-05-19

**Authors:** Zhen Yang, Min Wang, Yuchen Zhang, Fei Cai, Botao Jiang, Wenliang Zha, Wei Yu

**Affiliations:** ^1^Department of Pharmacology, School of Pharmacy, Hubei University of Science and Technology, Xianning, China; ^2^Hubei Province Key Laboratory on Cardiovascular, Cerebrovascular, and Metabolic Disorders, Hubei University of Science and Technology, Xianning, China; ^3^Department of Urology, Xianning Central Hospital, Xianning, China; ^4^Department of Surgery, Clinic Medical College, Hubei University of Science and Technology, Xianning, China; ^5^National Demonstration Center for Experimental General Medicine Education, Hubei University of Science and Technology, Xianning, China

**Keywords:** diabetic cardiomyopathy, prokineticin 2, prokineticin 2 receptors, AKT/GSK3β signaling pathway, apoptosis

## Abstract

Diabetic cardiomyopathy (DCM) is a complication of diabetes that can cause damage to myocardial structure and function. Metformin (Met) is a widely used type 2 diabetes treatment drug that exerts cardioprotective effects through multiple pathways. Prokineticin 2 (PK2) is a small-molecule secreted protein that plays pivotal parts in cardiomyocyte survival and angiogenesis. However, the role of Met in regulating the PK2 signaling pathway in DCM remains unclear. This experiment explored the effects of Met on high glucose (HG)-induced injury through the PK2/PKR pathway *in vivo* and *in vitro*. Cardiomyocytes isolated from adult or AKT-knockout mice were treated with HG (33 mmol/L) and PK2 or AKT1/2 kinase inhibitor (AKT inhibitor). Heart contraction properties based on cell shortening were evaluated; these properties included the resting cell length, peak shortening (PS), maximum speed of shortening/relengthening (±dL/dt), time to 90% relengthening (TR_90_), and time to peak shortening (TPS). Mice with streptozotocin-induced diabetes were treated with Met to evaluate cardiac function, myocardial structure, and the PK2/PKR and AKT/GSK3β pathways. Moreover, H9c2 cardiomyocytes were exposed to HG in the absence or presence of Met with or without the PK2 antagonist PKRA7 or the AKT inhibitor, and apoptotic proteins such as Bax and Bcl-2 and the PK2/PKR and AKT/GSK3β pathways were evaluated using western blot analysis. The prolongation of TR_90_ and decreases in PS and ±dL/dt caused by HG were ameliorated by PK2 in cardiomyocytes, but the effects of PK2 were ameliorated or negated by the AKT inhibitor and in AKT-knockout mice. Diabetic mice showed metabolic abnormalities, aberrant myocardial enzyme levels, declines in myocardial systolic and diastolic function associated with myocardial fibrosis, and pronounced apoptosis, but these effects were greatly rescued by Met treatment. Moreover, PK2, PKR1, and PKR2 expression and p-AKT/AKT and p-GSK3β/GSK3β ratios were decreased in diabetic mice, and these decreases were attenuated by Met. Likewise, H9c2 cells exposed to HG showed reduced PK2/PKR expression and decreased p-AKT/AKT and p-GSK3β/GSK3β ratios, and these effects were nullified by Met. In addition, the effects of Met on cardiomyocytes exposed to HG were abolished after intervention with PKRA7 or the AKT inhibitor. These results suggest that Met can activate the PK2/PKR-mediated AKT/GSK3β pathway, thus improving cardiac function and alleviating apoptosis in DM mice.

## Introduction

Diabetes mellitus (DM) is a metabolic disease caused by a combination of genetic and environmental factors that is characterized by persistent hyperglycemia and insulin deficiency. Currently, the diabetic population is becoming younger, and the International Diabetes Federation has reported that the total number of people with diabetes is expected to reach 642 million by 2040 (von Scholten et al., 2015; Ogurtsova et al., 2017). Diabetes is a strong risk factor for cardiovascular morbidity and mortality (Agrawal et al., 2006). Nowadays, the combinations of traditional Chinese medicines and modern Western medicine are widely used in clinical treatment to prevent or ameliorate the development of diabetic complications (Ceylan Isik et al., 2008). Diabetic cardiomyopathy (DCM) is a structural and functional disorder of the heart caused by diabetes (Rubler et al., 1972). Alterations in mitochondrial, apoptotic, intracellular Ca^2+^ anomalies, oxidative stress, as well as the expression of p-AKT, GSK3β all lead to damaged myocardial structure and function in diabetics (Wold et al., 2005; Zhang et al., 2012). In the current work, the specific or tissue selective PI3K modulators and AKT activation were considered as the potential treatment targets of DCM (Bi et al., 2020). However, the mechanisms underlying DCM remain unclear. Therefore, clinical studies are urgently needed to explore these mechanisms.

Prokineticin 2 (PK2) is an 8-kDa small-molecule secreted protein (Mollay et al., 1999). PK2 belongs to the prokineticin protein family, which participates in many biological processes, such as angiogenesis, hematopoiesis, and inflammation, by activating downstream signaling pathways (Martin et al., 2011; Maftei et al., 2014; Kurebayashi et al., 2015; Arora et al., 2016; Gordon et al., 2016). PK2 participates in myocardial diseases by binding to two agonist receptors, prokineticin receptor 1 (PKR1) and prokineticin receptor 2 (PKR2) (Negri et al., 2009). Insulin resistance, cardiac lipid deposition, interstitial fibrosis, and myocardial systolic diastolic function have been observed in PKR1-knockout mice (Boulberdaa et al., 2011a; Dormishian et al., 2013). In addition, AKT plays crucial roles in cardiac growth, coronary angiogenesis, and metabolic regulation (De Los Santos et al., 2017; Qi et al., 2017; Landa-Galvan et al., 2020). Recently, Su et al. (2020) demonstrated that PK2 relieves hypoxia/reoxygenation-induced injury in H9c2 cardiomyocytes by activating the AKT pathway. In our recent study, the role of the PK2-mediated AKT/GSK3β pathway in the development of DCM was investigated.

Metformin (Met) is a well-known therapeutic drug for diabetes. Met reduces blood glucose mainly by activating AMP-activated protein kinase (AMPK) and non-AMPK pathways. Met is thought to exert its primary antidiabetic action through the suppression of hepatic glucose production (Foretz et al., 2019). Met is also used in the treatment of diabetes complications such as DCM. Metformin ameliorates metabolic disorders, reduces ROS production and cardiac cell death, and improves myocardial structure and function on DCM (Sanit et al., 2019; Yang et al., 2019). Met also protects against brain and liver injury by activating AKT phosphorylation (Naicker et al., 2016; Xu et al., 2016). However, whether Met exerts a positive effect on DCM by regulating the AKT/GSK3β signaling pathway via PK2 is still unclear. Therefore, this study was conducted to elucidate the effects and the possible mechanisms of action of Met in high glucose (HG)-treated cardiomyocytes and DM mice myocardium.

## Materials and Methods

### Animals

Male C57BL/6J mice (22 ± 2 g, 5–6 weeks of age) were purchased from Jinan Pengyue Experimental Animal Breeding Co., Ltd. (China). AKT-knockout mice were provided by the University of Wyoming (Laramie, WY, United States). The animal protocols were in accordance with the Guide for the Care and Use of Laboratory Animals, and the use of the animals was approved by the Institutional Animal Care and Use Committee at Hubei University of Science and Technology.

### Cardiomyocyte Isolation and Mechanical Analyses

Hearts from adult wild-type or AKT-knockout mice were immediately perfused and then digested for 15 min. Then, the left ventricles were removed, and Ca^2+^ was added to a concentration of 1.25 mmol/L. The cardiomyocytes were used for shortening experiments. The mechanical properties of myocytes were evaluated with an IonOptix SoftEdge system. The resting cell length, time to peak shortening (TPS), peak shortening (PS), time to 90% relengthening (TR_90_), and maximum speed of shortening/relengthening (±dL/dt) were measured to evaluate cell shortening and relengthening after stimulation at 0.5 Hz (Aberle et al., 2004). The cells were incubated with Krebs–Henseleit bicarbonate solution containing HG (33 mmol/L) with or without PK2 (10 nmol/L, Sigma) for 4 h to observe the effect of PK2 on cardiac functional impairment induced by HG.

### Experimental DM Model Establishment and Met Treatment

Mice were randomized into three groups: a control group (*n* = 20), a DM group (*n* = 25), and a DM-Met group (*n* = 25). The mice in the DM group and the DM-Met group were injected intraperitoneally with 50 mg/kg/day streptozotocin (STZ, Sigma, United States) for five consecutive days. The control mice were administered with citrate buffer. Mice with blood glucose levels higher than 16.7 mmol/L were considered diabetic. The mice in the DM-Met group were given 250 mg/kg/day Met (Sino-American Shanghai Squibb Pharmaceuticals Ltd., China) in drinking water for 16 weeks.

### Cell Culture and Treatment

The H9c2 cell line was purchased from the China Center for Type Culture Collection (CCTCC, China), and the cells were cultured and maintained in Dulbecco’s modified Eagle’s medium (DMEM, HyClone, United States) supplemented with fetal bovine serum (FBS, 10%, Gibco, United States), penicillin (1%, Gibco), and streptomycin at 37°C in a humidified atmosphere (5% CO_2_ and 95% air). H9c2 cells were incubated with normal glucose (NG, 5.5 mmol/L) or HG (33 mmol/L) for 72 h with or without Met (2.5 mmol/L, Meilunbio, China), a PK2 antagonist (PKRA7, 10 μmol/L, Sigma), and an AKT1/2 kinase inhibitor (AKT inhibitor, 10 μmol/L, Sigma).

### Echocardiographic Assessment

Mice were anesthetized with isoflurane. Each rat was fixed on a rat plate, and the chest area was shaved. Cardiac function was evaluated using a two-dimensional guided M-mode echocardiography. Basic hemodynamic parameters, such as the heart rate (HR), left ventricular ejection fraction (LVEF), left ventricular fractional shortening (LVFS), left ventricular posterior wall thickness in diastole (LVPWD), and left ventricular internal dimension in end-diastole (LVIDD), were measured and calculated.

### Histopathologic Analysis

Cardiac tissue was fixed in 4% paraformaldehyde for 24 h and then embedded and sectioned. The sections were stained with hematoxylin and eosin (HE) and Masson’s trichrome. Myocardial microstructure was observed under a light microscope. Additional cardiac tissue was fixed in electron microscopy fixative and embedded in an acetone-812 embedding agent. The tissues were cut into 60-nm-thick sections and stained with uranyl acetate and lead citrate. Myocardial ultramicrostructure was observed by electron microscopy.

### Biochemical Indicators

Blood was collected from each group of animals, stored at 4°C overnight, and centrifuged at 3000 r/min for 15 min at 4°C in a low-temperature ultracentrifuge. The levels of lactic dehydrogenase (LDH), aspartate aminotransferase (AST), creatine kinase (CK), total cholesterol (TC), and triglyceride (TG) in the serum were tested according to procedures from the Nanjing Jiancheng Institute of Biological Engineering (China).

### Real-Time Quantitative Fluorescence PCR Analysis

Total RNA was extracted from heart tissues with TRIzol reagent. Reverse transcription was performed to synthesize cDNA. The samples were incubated at 65°C for 5 min and then cooled on ice. Reaction buffer (5×), mixed dNTPs, RiboLock RNase inhibitor, and RevertAid M-MuLV reverse transcriptase were sequentially added. The samples were incubated at 42°C for 60 min in a PCR instrument, and the reverse transcriptase was inactivated at 70°C for 5 min. The PCR amplification reaction conditions were as follows: 95°C for 10 min, 95°C for 15 s, and annealing at 60°C for 60 s for 40 cycles. Melting curve analysis was performed from 60°C to 95°C in 0.3°C increments over 15 s. The results were analyzed by the ΔΔCT method. Table 1 shows the primer sequences used for the PCR experiment.

**TABLE 1:** Primer sequences.

Primers	Forward sequence	Reverse sequence
PKR1	5′-GGCTTCCAGACAGAGCAG ATC-3′	5′-GACAGTCACAAAGCAGA GCGTA-3′
PKR2	5′-CTACTTCCTCTTCGTCTT CGGG-3′	5′-AGAAGTCTCGCACTATGG TAAAGC-3′

### Immunohistochemistry

After dewaxing and hydration of the conventionally prepared paraffin slices, the samples were incubated with primary antibodies (working concentration: 1:1000) at 4°C for 12 h and then incubated with secondary antibodies for 1 h. A solution of diaminobenzidine was added dropwise, and the slices were observed under a microscope. The procedure was carried out according to the kit instructions.

### TUNEL Assay

Apoptosis was tested with a terminal deoxynucleotidyl transferase-mediated dUTP-biotin nick end labeling (TUNEL) assay in paraffin-embedded heart sections. Ten fields were analyzed for each slice, and the number of positive cells among cardiomyocytes was determined for each field. The apoptosis rates were calculated as the percentages of positive cells (Long et al., 2018).

### Western Blot Analysis

Fifty milligrams of myocardial tissue was weighed, and RIPA lysis buffer was added. A BCA Protein Assay Kit (Beyotime, China) was used to test the protein concentration. Protein (50 mg) was added to each lane of a 12% SDS-polyacrylamide gel, and the proteins were separated and transferred to a polyvinylidene fluoride (PVDF) membrane. The membrane was blocked and incubated overnight at 4°C with AKT, p-AKT, GSK3β, p-GSK3β, Bax, Bcl-2 (1:1000, Cell Signaling Technology, United States), PK2 (1:1000, Abcam, United States), PKR1, and PKR2 (1:2000, Santa Cruz) antibodies. After incubation with secondary antibodies for an hour, the analysis was completed using an Enhanced Chemiluminescence Kit (Meilunbio, China).

### Statistical Analysis

The data are presented as the mean ± standard error of the mean (SEM). *T*-tests or one-way ANOVA was performed for all statistical analyses. *T*-tests were applied to analyze differences between two groups, and one-way ANOVA (Student–Newman–Keuls) was used to analyze differences among three or more groups. A *P* value less than 0.05 was considered to indicate statistical significance.

## Results

### Effect of PK2 on Cardiomyocyte Shortening

**FIGURE 1:**
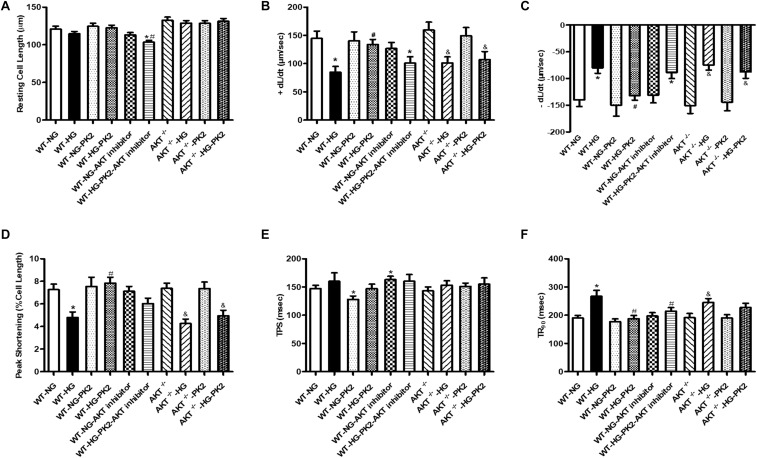
Effect of PK2 on cardiomyocyte shortening. WT, wild-type mouse cardiomyocytes; AKT^–/–^, AKT-knockout mouse cardiomyocytes; NG, normal glucose; HG, high glucose; NG-PK2, normal glucose plus PK2; HG-PK2, high glucose plus PK2. **(A)** Resting cell length. **(B)** +dL/dt. **(C)** –dL/dt. **(D)** PS. **(E)** TPS. **(F)** TR_90_. The data are mean ± SEM; * *P* value less than 0.05 versus the WT-NG; ^#^*P* value less than 0.05 versus the WT-HG; ^&^P value less than 0.05 versus the AKT^–/–^ group *n* = 35–75 cells per group.

As shown in Figure 1, compared with NG exposure, HG (33 mmol/L) exposure decreased the ±dL/dt and PS and prolonged the TR_90_ significantly but did not affect the resting cell length or TPS. PK2 significantly alleviated the HG-induced abnormalities in PS, ±dL/dt, and TR_90_ but had no effect on TPS or resting cell length. The effects were ameliorated or negated in AKT-knockout mouse cardiomyocytes and by AKT inhibitor treatment. These data strongly indicate that PK2 plays a pivotal role in the diabetic heart and that the important roles of AKT in PK2 provide benefits against glucose toxicity.

### Met Improved General Features and Echocardiographic Properties in DM Mice

Body weight (BW), heart weight (HW), and blood glucose levels reflect the general health state of mice. The results showed that BW and HW were significantly decreased and that the heart-to-body weight ratio (HW/BW) was significantly increased in the DM group compared with the control group; these changes were overtly reversed by Met treatment (Table 2). In addition, diabetic mice displayed significantly higher blood glucose levels than control mice. Treatment with Met improved the blood glucose levels in diabetic mice but did not restore them to normal levels. We have published the data of BW and blood glucose (Liu et al., 2019).

**TABLE 2:** Met improved general features and echocardiographic properties in DM mice.

Parameter	control	DM	DM-Met
HW (mg)	144.9 ± 2.8	99.9 ± 6.3*	111.6 ± 4.9*
HW/BW (mg/g)	4.6 ± 0.1	5.2 ± 0.2*	4.0 ± 0.2*^#^
HR (bpm)	505.21 ± 36.84	372.71 ± 34.22*	462.25 ± 6.24
LVPWD (mm)	0.68 ± 0.06	0.72 ± 0.04	0.58 ± 0.01
LVIDD (mm)	3.38 ± 0.12	3.57 ± 0.06	3.43 ± 0.05
LVEF (%)	72.13 ± 3.17	58.31 ± 4.18*	71.32 ± 2.64^#^
LVFS (%)	40.64 ± 2.66	30.37 ± 2.75*	39.72 ± 2.24^#^

HR, heart rate; LVPWD, left ventricular posterior wall thickness; LVIDD, left ventricular internal dimension in end-diastole; LVEF, left ventricular ejection fraction; LVFS, left ventricular fractional shortening. The data are mean ± SEM; *P value less than 0.05 versus the control group; ^#^P value less than 0.05 versus the DM group; n = 4–11 per group.

Echocardiography is a technique for examining the anatomy and function of the heart. Echocardiographic evaluation revealed that LVPWD and LVIDD were slightly increased in DM mice, but these increases were mitigated by Met. Moreover, HR was significantly reduced in DM mice, and the reduction was partly eliminated by Met. Moreover, DM mice showed remarkable reductions in both LVEF and LVFS that were notably nullified by Met (Table 2).

### Met Protected Against Myocardial Injury in DM Mice

**FIGURE 2:**
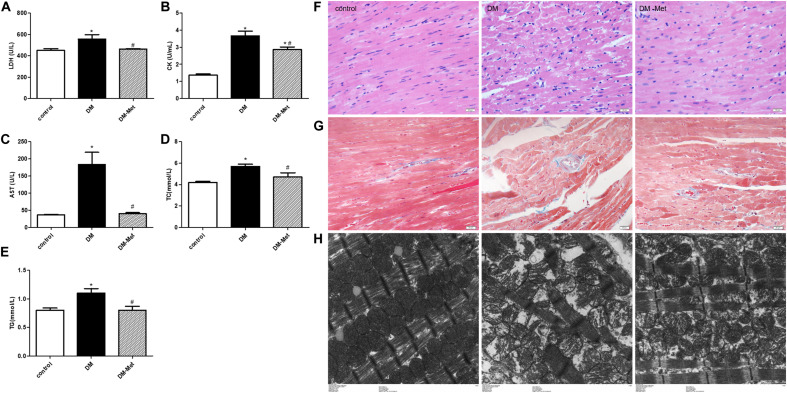
Met protected against myocardial injury in DM mice. **(A)** LDH, **(B)** CK, **(C)** AST, **(D)** TC, and **(E)** TG levels. *n* = 8–22 per group. **(F)** HE staining of cardiomyopathy tissue. **(G)** Masson’s trichrome staining of cardiomyopathy tissue. **(H)** Transmission electron microscopy images of the left ventricle. The data are mean ± SEM; * *P* value less than 0.05 versus the control group; ^#^ *P* value less than 0.05 versus the DM group; magnification = 400×; scale bar = 20 μm; *n* = 5 per group.

Myocardial zymograms are recognized as effective indicators of cardiac function. The levels of LDH, CK, and AST in serum were significantly increased in the DM group compared to the control group, the effects of which were reversed by Met administration (Figures 2A–C). Moreover, TC and TG levels were significantly elevated in DM mice, indicating that DM mice had abnormal blood lipid metabolism, the effects of which were ameliorated by Met (Figures 2D,E).

Hematoxylin and eosin staining showed that the myocardial cells in the control group were clearly visible and arranged neatly, while those in the DM group showed disordered myocardial fiber arrangement and vacuolization. Interestingly, the myocardial fibers were denser in the Met treatment group than in the DM group, and less vacuolization was observed (Figure 2F).

Masson’s trichrome staining showed that there was less blue collagen in the myocardial interstitium in the control group than in the other groups. In contrast, increased interstitial fibrosis was evident in the DM mice hearts, and the fibrotic changes were significantly attenuated after Met administration (Figure 2G).

Electron microscopy revealed that in the control group, the mitochondria were intact, the myofilaments were neat and complete, and the Z-line and M-line were clear. However, in the DM group, the myocardial cells were arranged in a disorderly manner, the mitochondria were swollen, and myofilament fracture was present. Met ameliorated these abnormalities, causing the myofilament arrangement to be more regular and the mitochondrial structure to be more complete and reducing myofilament fracture (Figure 2H).

### Met Prevented Diabetes-Induced Cardiomyocyte Apoptosis

**FIGURE 3:**
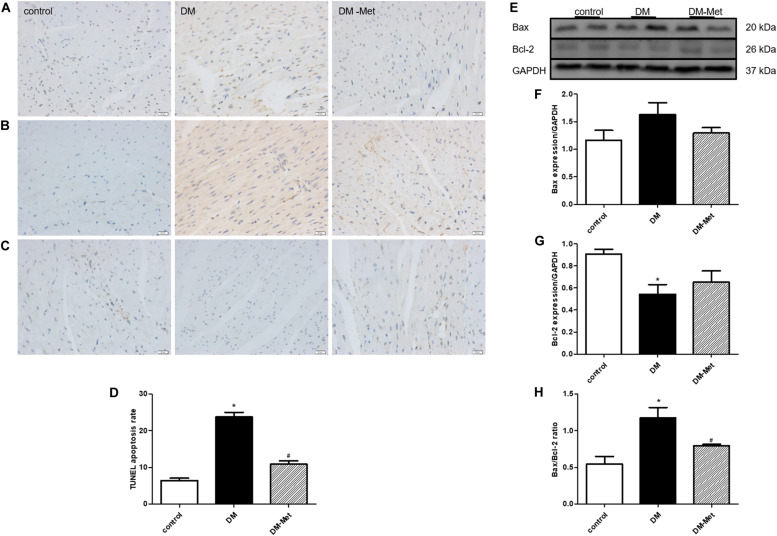
Met prevented diabetes-induced cardiomyocyte apoptosis. **(A)** TUNEL staining. **(B)** Immunohistochemical staining for Bax. **(C)** Immunohistochemical staining for Bcl-2. **(D)** Analysis of TUNEL-positive cells. **(E)** Images of Bcl-2 and Bax protein levels. **(F)** Analysis of Bax. **(G)** Analysis of Bcl-2. **(H)** Analysis of the Bax/Bcl-2 ratio. The data are mean ± SEM; * *P* value less than 0.05 versus the control group; ^#^ *P* value less than 0.05 versus the DM group; *n* = 3–5 per group.

TUNEL staining was used to detect apoptotic cardiomyocytes. The data showed that there were many more apoptotic cardiomyocytes (stained in brown) in the DM group than in the control group, but Met treatment significantly decreased DM-induced myocardial cell apoptosis (Figures 3A,D).

Apoptosis-related proteins play important roles in apoptosis progression. We thus carried out a more in-depth experiment to investigate whether apoptosis-related proteins affected the amelioration of DM-induced cardiomyocyte apoptosis by Met. As shown in Figures 3B,C, Bax protein upregulation and Bcl-2 protein downregulation (as assessed using immunohistochemical staining) were evident in the DM group compared with the control group, and these effects were reversed by Met. Consistent with the immunohistochemical staining results, the western blot results showed that Bax expression was enhanced in the DM group, whereas Bcl-2 expression was remarkably reduced; these changes were partly attenuated by Met administration. Furthermore, the Bax/Bcl-2 ratio was elevated in the DM group compared to that in the control group, the effect of which was markedly abrogated by Met administration (Figures 3E–H).

### Met Increased the Expression of PK2/PKR Signaling Pathway Members in DM Mice

**FIGURE 4:**
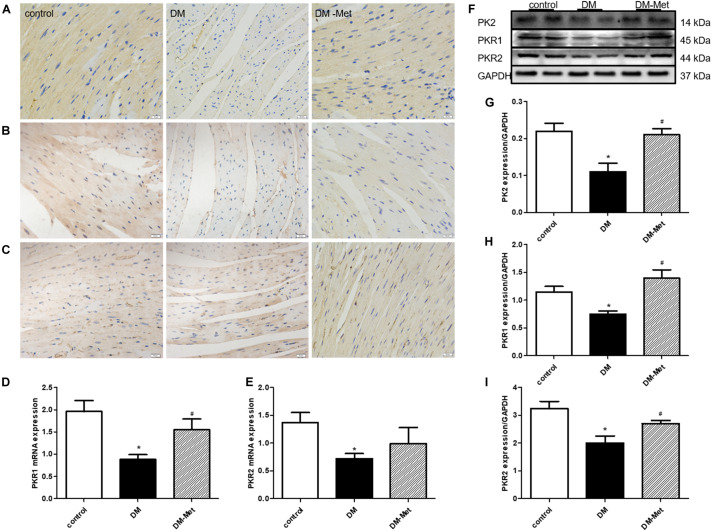
Met increased the expression of PK2/PKR signaling pathway members in DM mice. **(A)** Immunohistochemical staining for PK2. **(B)** Immunohistochemical staining for PKR1. **(C)** Immunohistochemical staining for PKR2. *n* = 4–5 per group. **(D)** Expression of PKR1 mRNA, as determined by RT-PCR. **(E)** Expression of PKR2 mRNA, as determined by RT-PCR. **(F)** Images of PK2, PKR1, and PKR2 protein levels. **(G)** Analysis of PK2. **(H)** Analysis of PKR1. **(I)** Analysis of PKR2. The data are mean ± SEM; * *P* value less than 0.05 versus the control group; ^#^ *P* value less than 0.05 versus the DM group; *n* = 4–8 per group.

Previous studies have suggested that PK2 plays essential roles in the survival, proliferation, and apoptosis of cardiomyocytes. To further elucidate the role of PK2 in Met-mediated cardioprotection, we measured the effects of Met on PK2/PKR signaling pathway members. The results of immunohistochemical staining showed that the expression of PK2, PKR1, and PKR2 (brown labeling) was lower in the DM group than in the control group, the effects of which were ablated by Met treatment (Figures 4A–C). Constant to immunohistochemical results, the levels of PKR1 and PKR2 mRNA were also significantly downregulated, the effects of which were partly or considerably reversed by Met treatment (Figures 4D,E). Furthermore, the western blot results showed that the levels of PK2, PKR1, and PKR2 protein were markedly lower in the DM group than in the control group, but higher in the DM-Met group than in the DM group (Figures 4F–I). The data suggest that Met exerts a positive effect against diabetes-induced cardiac injury primarily by increasing the expression of PK2/PKR pathway members.

### Met Activated the AKT/GSK3β Signaling Pathway in DM Mice

**FIGURE 5:**
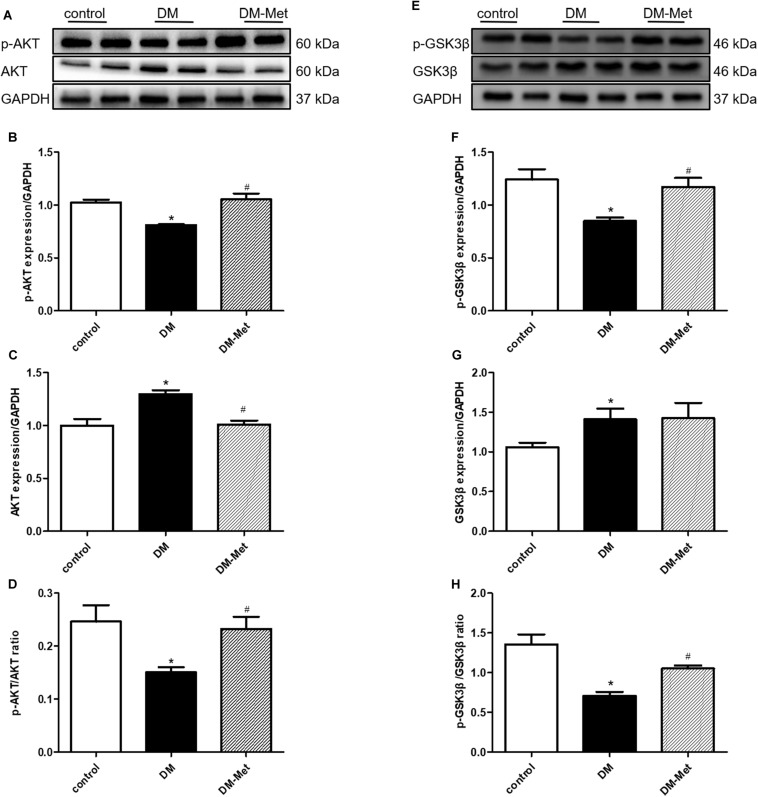
Met activated the AKT/GSK3β signaling pathway in DM mice. **(A)** Images of p-AKT and AKT protein levels. **(B)** Analysis of p-AKT. **(C)** Analysis of AKT. **(D)** Analysis of the p-AKT/AKT ratio. **(E)** Images of p-GSK3β and GSK3β protein levels. **(F)** Analysis of p-GSK3β. **(G)** Analysis of GSK3β. **(H)** Analysis of the p-GSK3β/GSK3β ratio. The data are mean ± SEM; * *P* value less than 0.05 versus the control group; ^#^ *P* value less than 0.05 versus the DM group; *n* = 4 per group.

Previously obtained evidence has revealed that the cardioprotective actions of PK2/PKR may involve the phosphorylation of AKT, and impaired AKT activity in response to insulin is a common feature of DM (Wang C.Y. et al., 2016; Su et al., 2020). We therefore assessed the effect of Met on the AKT/GSK3β signaling pathway. As shown in Figure 5, the p-AKT/AKT and p-GSK3β/GSK3β ratios were significantly declined in the DM group, the effects of which were notably reversed by Met treatment.

### Effects of Met on the PK2/PKR Signaling Pathway in HG-Treated Cardiomyocytes

**FIGURE 6:**
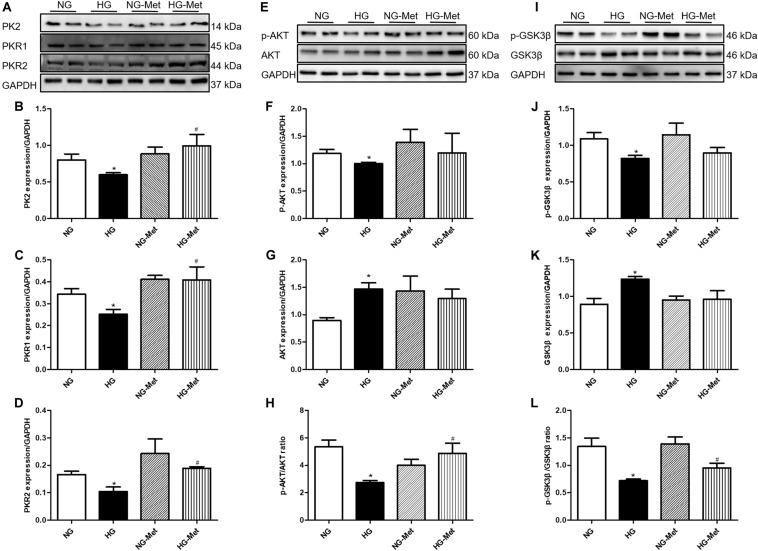
Effects of Met on the PK2/PKR and AKT/GSK3β signaling pathways in HG-treated cardiomyocytes. NG-Met, normal glucose plus metformin; HG-Met, high glucose plus metformin. **(A)** Images of PK2, PKR1, and PKR2 protein levels. **(B)** Analysis of PK2. **(C)** Analysis of PKR1. **(D)** Analysis of PKR2. **(E)** Images of p-AKT and AKT protein levels. **(F)** Analysis of p-AKT. **(G)** Analysis of AKT. **(H)** Analysis of the p-AKT/AKT ratio. **(I)** Images of p-GSK3β and GSK3β protein levels. **(J)** Analysis of p-GSK3β. **(K)** Analysis of GSK3β. **(L)** Analysis of the p-GSK3β/GSK3β ratio. The data are mean ± SEM; * *P* value less than 0.05 versus the NG group; ^#^ *P* value less than 0.05 versus the HG group; *n* = 3–4 per group.

To further validate the role of the PK2/PKR pathway in Met-induced cardiomyocyte mechanical responses against HG *in vitro*, the PK2/PKR signaling pathway was examined using western blot analysis. The data showed that PK2, PKR1, and PKR2 expression levels were overtly decreased in H9c2 cardiomyocytes exposed to HG, but the changes were significantly abrogated by Met treatment (Figures 6A–D).

### Met Activated the AKT/GSK3β Signaling Pathway in HG-Treated Cardiomyocytes

To determine whether Met activates the PK2-mediated AKT/GSK3β signaling pathway in HG-treated cardiomyocytes, AKT and GSK3β signaling was examined using western blot analysis. As demonstrated in Figures 6E–L, HG incubation markedly decreased the ratio of p-AKT/AKT and p-GSK3β/GSK3β, the effects of which were negated by Met treatment.

### A PK2 Antagonist and an AKT Inhibitor Blocked the Effects of Met on HG-Treated Cardiomyocytes

**FIGURE 7:**
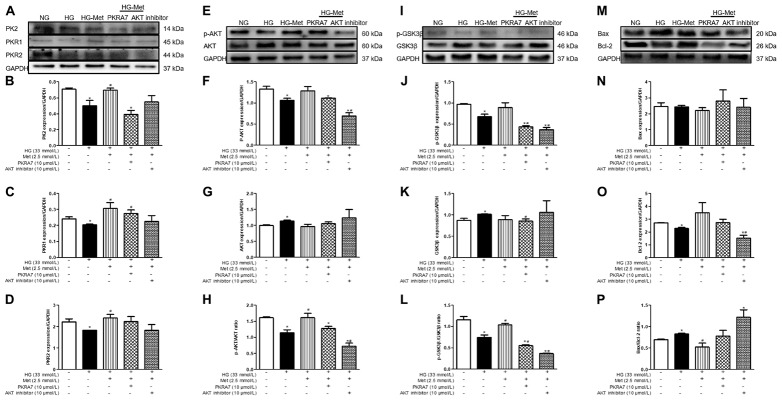
A PK2 antagonist and an AKT inhibitor blocked the effects of Met on HG-treated cardiomyocytes. **(A)** Images of PK2, PKR1, and PKR2 protein levels. **(B)** Analysis of PK2. **(C)** Analysis of PKR1. **(D)** Analysis of PKR2. **(E)** Images of p-AKT and AKT protein levels. **(F)** Analysis of p-AKT. **(G)** Analysis of AKT. **(H)** Analysis of the p-AKT/AKT ratio. **(I)** Images of p-GSK3β and GSK3β protein levels. **(J)** Analysis of p-GSK3β. **(K)** Analysis of GSK3β. **(L)** Analysis of the p-GSK3β/GSK3β ratio. **(M)** Images of Bcl-2 and Bax protein levels. **(N)** Analysis of Bax. **(O)** Analysis of Bcl-2. **(P)** Analysis of the Bax/Bcl-2 ratio. The data are mean ± SEM; * *P* value less than 0.05 versus the NG group; ^#^ *P* value less than 0.05 versus the HG group; *n* = 3–4 per group.

To clarify whether PK2 and PK2-mediated AKT play key roles in the Met-induced beneficial response to HG challenge, cardiomyocytes were incubated with NG or HG medium in the absence or presence of Met and PKRA7 or an AKT inhibitor. As shown in Figures 7A–P, incubation with PKRA7 partially or overtly reversed the Met-induced increases in PK2, PKR1, and PKR2 expression. In parallel, the Met-induced elevations in the p-AKT/AKT and p-GSK3β/GSK3β ratios were abolished by treatment with PKRA7. In addition, the AKT inhibitor effectively eliminated the effects of Met on the p-AKT/AKT and p-GSK3β/GSK3β ratios and partially attenuated the effects of Met on PK2 and PKR expression. Furthermore, Met failed to regulate the expression of Bcl-2 and Bax when PKRA7 or the AKT inhibitor was applied. These results illustrate that Met exerts protective effects against glucose toxicity through the PK2/PKR-mediated AKT/GSK3β pathway.

## Discussion

The salient findings from our current study suggest that the PK2/PKR pathway plays a crucial role in the pathogenesis of DCM and that Met treatment prevents diabetes-induced glucose and lipid metabolism dysfunction, cardiomyocyte apoptosis, fibrosis, and cardiac insufficiency by stimulating PK2/PKR and the downstream AKT/GSK3β pathway. Glucose toxicity is considered to be the key factor in cardiovascular complications caused by diabetes, but the clinical treatment of DCM is still challenging. Our data reveal a likely role for the PK2/PKR pathway in the anti-DCM effects of Met.

It is well known that myocardial fibrosis and apoptosis contribute to the development of DCM (Gao et al., 2019). Several studies have also shown that left ventricular dysfunction and myocardial apoptosis are closely related, which indicates that inhibition of cardiomyocyte apoptosis can restore cardiac function (Yu et al., 2016). Various apoptotic stimuli, such as hyperglycemia and lipid metabolism dysfunction, can lead to enormous cardiomyocyte loss, which is proven to facilitate contractile dysfunction, fibrosis, and remodeling. Therefore, inhibition of cardiomyocyte fibrosis and apoptosis is thought to be a valid strategy for treating DCM. In the current work, disturbance of glucose and lipid metabolism, overt remodeling mainly presented as disorderly arrangement of myocardial cells, considerable deposition of collagen fibers in the myocardial interstitium, elevated HW/BW, and compromised cardiac function were observed in diabetic mice. However, long-term administration of Met rescued against DM-induced cardiac remodeling and functional anomalies. Notably, apoptosis-related Bcl-2 family members regulate apoptosis progression. Bax and Bcl-2 are members of the Bcl-2 family, and Bax binds with Bcl-2 to block upstream apoptotic signal transduction and promote cell survival and growth (Thandavarayan et al., 2011; Jafri et al., 2019). We discovered diabetes triggered cardiomyocytes apoptosis and increased Bax/Bcl-2 ratios in DM mice compared with control mice. However, Met reduced the diabetes-induced increases in cardiomyocyte apoptosis and the Bax/Bcl-2 ratio. Thus, it is plausible to speculate that repair of cardiac function upon Met treatment is conducive to the inhibition of cardiomyocyte apoptosis.

Prokineticin 2 expression has been observed in a variety of tissues, such as the brain, heart, and testes. PK2 affects various biological processes, including neuronal survival, olfactory bulb morphogenesis, testis development, and circadian rhythms (Boulberdaa et al., 2011b; Neal et al., 2018). In recent years, evidence has demonstrated that PK2 protects against oxidative stress in cardiomyocytes and H9c2 cells, suggesting that PK2 is inversely related to apoptosis (Gao et al., 2019). In addition, PK2 treatment can reduce food intake and BW in lean and obesity models (Gardiner et al., 2010). However, the precise functions of PK2 in diabetic-induced cardiac injury have not been determined to date. In this study, we found that PK2 ameliorated HG-induced cardiac contractile dysfunction by elevating PS and ±dL/dt and decreasing TR_90_ in cardiomyocytes, supporting that PK2 may be the key factor in DCM. Prokineticins exert multiple biological effects by activating PKR1 and PKR2 and mostly differ in their N-terminal sequences. Previous studies have revealed that PK2 and PKR1 levels are reduced in the late stage of heart failure, but PKR1 overexpression can protect cardiomyocytes from hypoxia injury-elicited apoptosis (Urayama et al., 2007; Gasser et al., 2015). Moreover, accumulating studies have demonstrated that PK2/PKR is associated with many types of cardiometabolic risk factors and exerts a vital role in cardiovascular disease development (Wang Y. et al., 2016). Upregulation of PK2 manipulates epicardium-derived progenitor cells involved in tissue repair/regeneration in heart diseases, whereas PKR1 knockout in the epicardium results in ventricular hypoplasia, ventricular septal defects, and even embryo necrosis (Curtis et al., 2013). Similarly, PK2 induces the growth of myocardium derived from mice, but the effect is abolished in PKR1-knockout myocardium (Gasser et al., 2015). In addition, PKR1-null mice exhibit cardiomyocyte apoptosis and contractile defects (Boulberdaa et al., 2011a). Recently, numerous studies have explored the cardioprotective effect of Met in the DCM myocardium, but whether Met can activate PK2 and its pathway in DCM remains unclear. Since our data showed that PK2 treatment protected against HG-induced cardiac function compromise *in vitro*, we made further efforts to investigate the protective effect of Met on HG-induced cardiac damage mediated via regulation of the PK2/PKR pathway *in vitro* and *in vivo*. As expected, our current results suggested that PK2, PKR1, and PKR2 expression was remarkably dampened both in diabetic mouse hearts and H9c2 cardiomyocytes exposure to HG, which further confirmed that a decrease in PK2 may be the main contributor to myocardial apoptosis in DCM. Interestingly, after administration of Met, PK2, PKR1, and PKR2 expression was significantly increased *in vivo* and *in vitro*. In addition, PKRA7 partly or considerably counteracted the beneficial effects of Met against glucose toxicity. Our observations favored a role for activation PK2/PKR pathway in the Met-offered beneficial mechanical effects against HG-induced cardiac damage.

Cumulative evidence suggests that PK2/PKR participates in myocardial survival, angiogenesis, and the hematopoietic system through the AKT and STAT3 signaling pathways (Urayama et al., 2007; Xin et al., 2013). AKT appears to regulate cell survival, growth, apoptosis, and angiogenesis. Abnormal AKT regulation leads to impaired glucose tolerance in diabetic patients (Chen et al., 2001). GSK3β, one of the downstream substrates of AKT, is involved in physiological processes including cell death (Wang et al., 2009). It is now recognized that glucose utilization and abnormal free fatty acid oxidation are involved in the development of DCM (Neglia et al., 2007). Thus, activating AKT and inhibiting GSK3β activity can be considered effective treatment strategies for DCM. Our study clearly shows that regulation of the PK2-mediated AKT/GSK3β pathway may be a potential mechanism of the cardioprotective role of Met. This possibility is supported by several pieces of experimental data. First, we found that the protective effect of PK2 against HG challenge was completely negated in AKT-knockout mice and AKT inhibitor-treated isolated cardiomyocytes. Second, the results revealed that the p-AKT/AKT and p-GSK3β/GSK3β ratios were notably decreased not only in diabetic mice but also in HG-challenged H9c2 cardiomyocytes, the effects of which were significantly reversed by Met treatment. Third, the effects of Met on cardiomyocytes with HG-induced injury, such as the Met-mediated promotion of AKT and GSK3β phosphorylation and the regulation of apoptosis-related protein expression, were abolished after treatment with an AKT inhibitor. Therefore, Met-mediated reductions in diabetes-induced apoptosis might be attributable to activation of the PK2-mediated AKT/GSK3β pathway.

### Limitations of the Experiment

Our current studies have elucidated the important role of PK2 signaling cascade in DCM and Met ameliorates HG-induced cardiac damage through the PK2-mediated AKT/GSK3β pathway using *in vivo* diabetes animal models and *in vitro* HG-exposed cardiomyocytes. Any conclusions about the exact role of the Met activated PK2-AKT signaling cascade in DCM must be taken with caution. As proof of concept evidence is still lacking from genetically engineered mouse models of PK2 and AKT, it is quite challenging to better understand the role of PK2 signaling in biological process.

In summary, our data suggest that PK2 may participate in pathophysiological changes in DCM and that Met protects against diabetes-induced cardiac and remodeling, apoptosis, and dysfunction via activation of the PK2/PKR signaling, thereby regulating the AKT/GSK3β pathway. These findings suggest new strategies for the prevention and treatment of DCM, which are worthy of further clinical study.

## Data Availability Statement

The raw data supporting the conclusions of this article will be made available by the authors, without undue reservation, to any qualified researcher.

## Ethics Statement

The animal study was reviewed and approved by the Institutional Animal Care and Use Committee at Hubei University of Science and Technology.

## Author Contributions

ZY, MW, YZ, FC, and BJ performed the experiments. WZ and WY contributed to analysis and interpretation of the data, and responsible for critically revising the manuscript. All authors read and approved the final manuscript.

## Conflict of Interest

The authors declare that the research was conducted in the absence of any commercial or financial relationships that could be construed as a potential conflict of interest.
